# Morphology-Controlled High-Efficiency Small Molecule Organic Solar Cells without Additive Solvent Treatment

**DOI:** 10.3390/nano6040064

**Published:** 2016-04-08

**Authors:** Il Ku Kim, Jun Hyung Jo, Jung-Ho Yun

**Affiliations:** 1 National Photonics Semiconductor Lab, National Photonics Semiconductor Inc., Suwon-Si 17113, Korea; 2 School of Information and Communication Technology, Griffith University, Southport QLD 4222, Australia; j.jo@griffith.edu.au; 3 Nanomaterials Centre, School of Chemical Engineering, University of Queensland, Brisbane QLD 4072, Australia; j.yun1@uq.edu.au

**Keywords:** small molecule, organic solar cell, bulk-heterojunction, optical simulation

## Abstract

This paper focuses on nano-morphology-controlled small-molecule organic solar cells without solvent treatment for high power-conversion efficiencies (PCEs). The maximum high PCE reaches up to 7.22% with a bulk-heterojunction (BHJ) thickness of 320 nm. This high efficiency was obtained by eliminating solvent additives such as 1,8-diiodooctane (DIO) to find an alternative way to control the domain sizes in the BHJ layer. Furthermore, the generalized transfer matrix method (GTMM) analysis has been applied to confirm the effects of applying a different thickness of BHJs for organic solar cells from 100 to 320 nm, respectively. Finally, the study showed an alternative way to achieve high PCE organic solar cells without additive solvent treatments to control the morphology of the bulk-heterojunction.

## 1. Introduction

During the last decades, bulk-heterojunction (BHJ) small-molecule organic solar cells (OSCs) have received more research attention [1,2,3,4,5,6,7,8,9]. BHJ OSCs have interpenetrating networks in a blend of conjugated organic donors and fullerene-derivative acceptors for their alternative potential in obtaining low-cost clean energy solutions [1,2,3,4,5,6]. Among the many research issues, surface morphology control is an essential part of the spin-coating process to form thin films to get high-efficiency OSCs [2]. In the BHJ photoactive layer, the ultrafast photo-induced charge transfer at the interface of the phase-separated acceptor and donor is also needed for the high performance of OSCs before charge recombination has happened [10,11,12]. However, despite polymeric BHJ OSCs reported with high power conversion efficiencies (PCEs), others have pointed out some drawbacks. For instance, the organic polymer has batch-to-batch variations, molecular weight differences, polydispersity and impurity which could lead to a major obstacle for high-performance organic solar cells [2,13,14,15]. To address the above issues, small-molecule BHJs (SM BHJs) were introduced [1,2,3,4,5,8].

In contrast, it has been reported that solution-processed small-molecule BHJ solar cells have a well-defined molecular structure with intermediate dimensions [1,2]. Examples of solution-processed SM BHJ OSCs with PCEs ranging from 6.7% to 8.01% have been reported with a 1,8-diiodooctane (DIO) additive solution [1,2,3,4]. These results were mainly obtained from the morphological control of the BHJ layer in terms of the additive solvent treatment. However, there are major concerns surrounding morphology control by using an additive solution such as the optimization of solution ratio control, and a limited number of applicable matching solutions (*i.e.*, chlorobenzene, 1,2-dichlorobenzene) [1,2,3,4]. In addition, the usage of a solvent additive affects unexpected solar cell performance. To avoid this, a study suggests the treatment of the active layer by drying speed or washout with methanol [16]. However, this is also not the fundamental method to solve the additional solvent problem issue. Therefore, it does not always guarantee a high efficiency of OSCs. Furthermore, strong aggregation is another problem during the spin-coating process used to produce a high quality of thin film for SM BHJs [2].

In this paper, we report solution-processed high-efficiency SM BHJ OSCs without a DIO additive solvent treatment. For that, a well-developed small-molecular donor (*p*-DTS(FBTTh_2_)_2_) will be applied [1,2,3,4,5]. This small-molecule donor has good characteristics of excellent solubility in organic solvents, strong optical absorption (600–800 nm) and a good hole mobility (≈0.1 cm^2^/Vs) [2]. Furthermore, a generalized transfer matrix method (GTMM) for the optical modeling analysis result will be introduced to confirm the behavior of light absorption in the BHJ photoactive layer of different thicknesses [17,18,19].

## 2. Experimental Section

**Figure 1:**
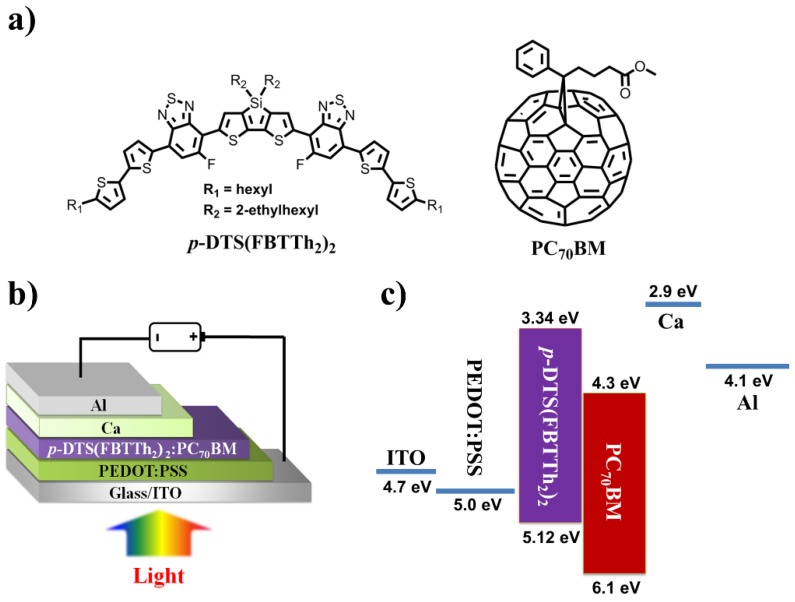
(**a**) Chemical structures of *p*-DTS(FBTTh_2_)_2_ and PC_70_BM; (**b**) device architecture for small-molecule bulk-heterojunction organic solar cells (SM BHJ OSCs); (**c**) band diagram of SM BHJ OSCs.

To address the particular issues mentioned above, OSCs fabricated through the incorporation of a donor-acceptor blend of *p*-DTS(FBTTh_2_)_2_ and [6,6]-phenyl-C_71_-butyric-acid-methyl-ester (PC_70_BM) to form a BHJ photoactive layer. Chemical structures for OSC showed in Figure 1a. *p*-DTS(FBTTh_2_)_2_ and PC_70_BM were dissolved in chlorobenzene (CB) and stirred over 24 h with a total concentration of 50 mg/mL. Indium tin oxide (ITO)-coated glass substrates were cleaned sequentially by ultrasonic treatment in Alconox detergent, deionized water, acetone and isopropyl alcohol. A polymeric conducting thin layer of PEDOT:PSS (40 nm) was spun-cast on top of the ITO-coated glass substrate. Before spin-coating of BHJ thin film, co-dissolved donor-acceptor blend was heated at 100 °C for 30 min. Then the *p*-DTS(FBTTh_2_)_2_:PC_70_BM BHJ layer was spin-cast from the heated and blended solution with different spin speed to form a different thickness of BHJs from 300 to 2000 rpm. During the solution preparation and film formation processes, 1,8-diiodooctane (DIO) additive solvent treatment was avoided. Finally, Ca (5 nm) and Al (100 nm) electrodes were deposited on top of BHJ photoactive layer (Figure 1b). Post-annealing process is also not applied for all devices. The energy band diagram of SM BHJ OSC showed in Figure 1c. Electrical characterization of all OSCs had done in the air after encapsulation. A commercial *J*-*V* and external quantum efficiency (EQE) characterization system was supplied by PV Measurement Inc. (Boulder, CO, USA) to obtain the data. A GTMM analysis was accomplished to calculate and analyse the multi-layered interface OSCs. For transfer matrix analysis, optical constants of all layers have been obtained by the ellipsometry method. Finally, the nano-morphology of BHJ film was investigated by atomic force microscopy (AFM).

## 3. Results and Discussion

**Figure 2:**
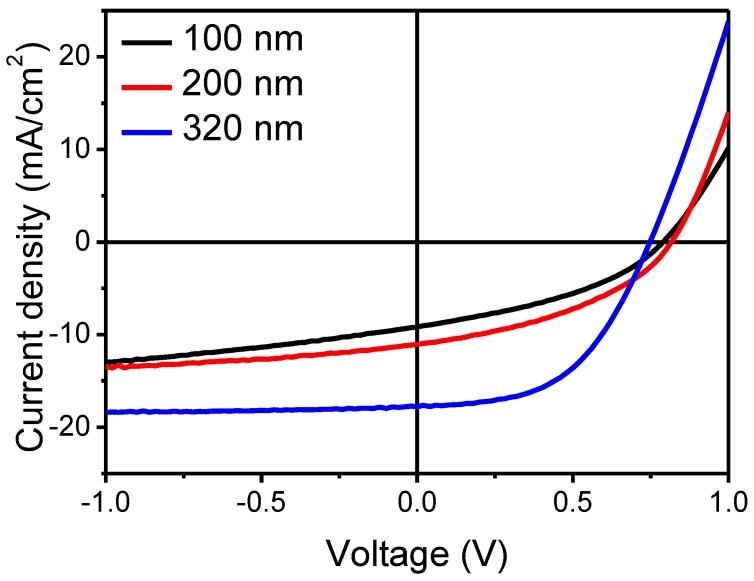
Measured density/voltage (*J*-*V*) curves of SM BHJ OSCs.

SM BHJ OSCs have been tested under simulated 100 mW/cm^2^ Air Mass (AM) 1.5G illumination. The optimized ratio of the small molecule donor (*p*-DTS(FBTTh_2_)_2_) to PC_70_BM was chosen to be the same as the reported ratio (60:40 *w/w*) [1]. Device current density/voltage (*J*-*V*) characteristics of SM BHJ OSCs, in terms of BHJ without DIO treatment, with different photoactive thicknesses are shown in Figure 2.

A *V*_oc_ as high as 0.74 V was observed in all devices. Combined with its high *J*_sc_ and fill factor (*FF*), a high power conversion efficiency (PCE) of 6.83% on average was measured with 320-nm-thick devices and the highest PCE was measured at 7.22% (*V*_oc_ = 0.74 V, *J*_sc_ = 18.23 mA/cm^2^, *FF* = 0.54). The measured PCE of our device (2.78%) is much greater than the reported PCE value (1.8%) with the same BHJ thickness of 100 nm [1]. It is strong evidence to confirm that an additive solvent such as DIO is not an essential part for controlling the device performance, while the majority of the research groups have focused on using an additive solvent to control the *p*-DTS(FBTTh_2_)_2_ and small molecule donor materials.

**Figure 3:**
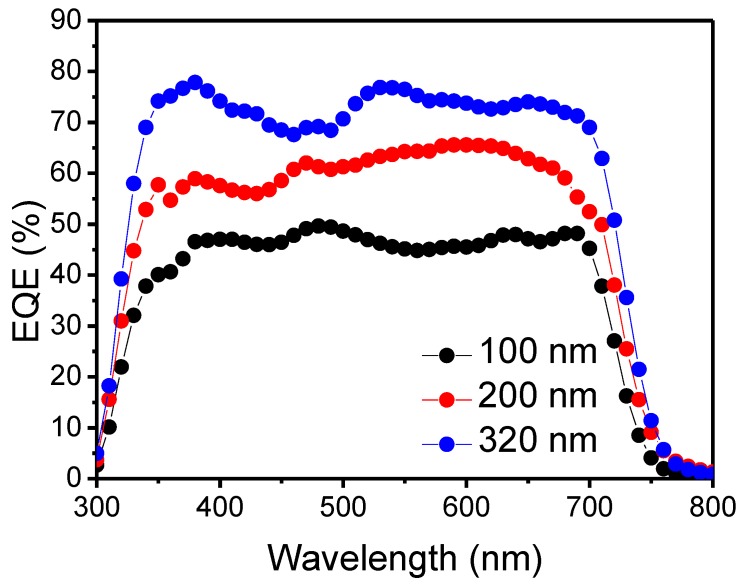
Measured external quantum efficiency (EQE) spectra of SM BHJ OSCs.

The EQE spectra were obtained to confirm the accuracy of the SM BHJ OSC’s photo-generated *J*-*V* result. The results are shown in Figure 3. Significantly, an EQE over 70% was achieved with a 320 nm SM BHJ photoactive layer from a wavelength range of 350–700 nm. A 1.5 G spectrum was applied to calculate the *J*_sc_ value by integrating the EQE data. The calculated *J*_sc_ values are in good agreement with the directly measured values. Furthermore, these EQE spectra confirm Beer-Lambert law by increasing the photoactive layer thicknesses. It is good to note that EQE spectra results obtained much better results to compare with the literature [1,3,4,8]. A summary of the SM BHJ OSCs’ performance is shown in Table 1.

**Figure 4:**
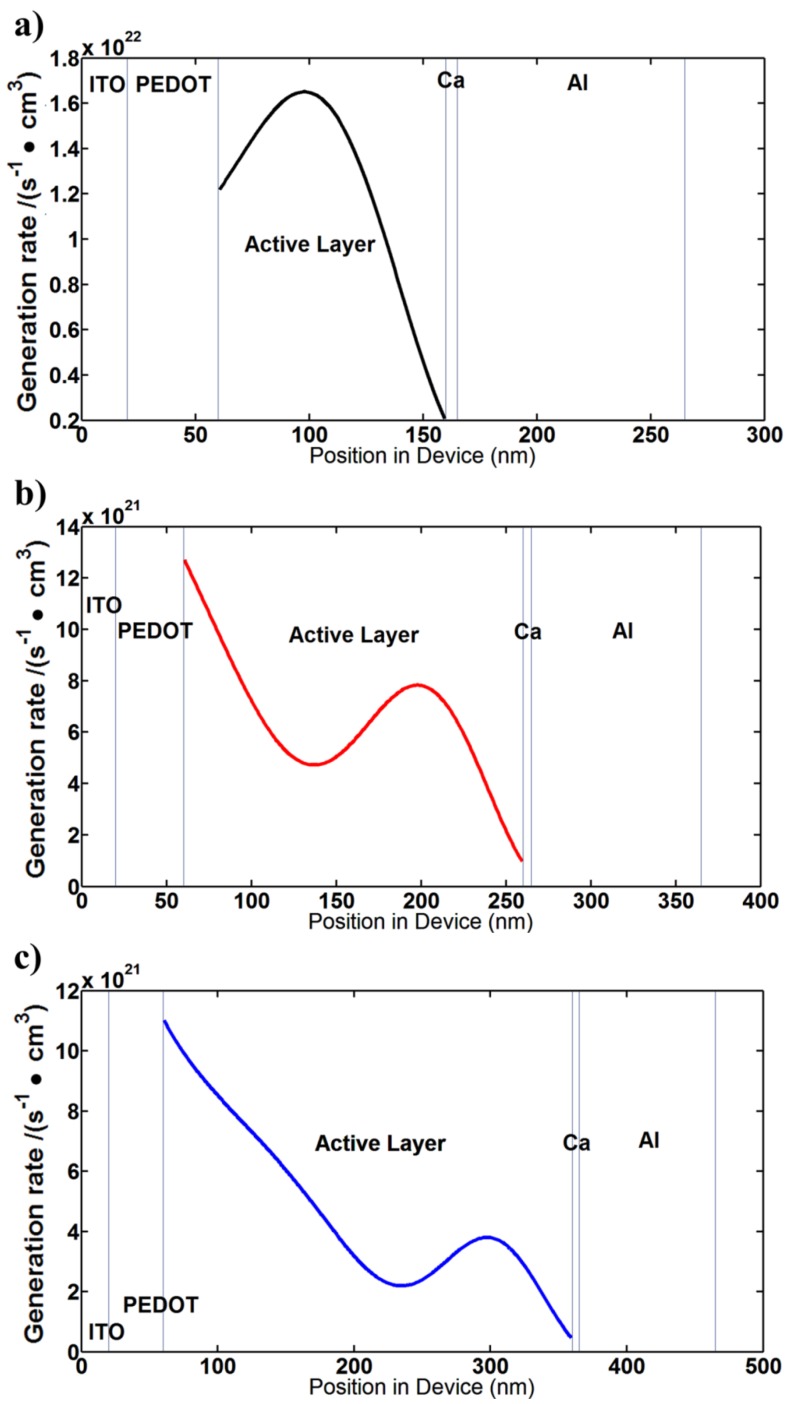
Simulation results of charge generation rates of SM BHJ OSCs: (**a**) BHJ active layer = 100 nm; (**b**) BHJ active layer = 200 nm; (**c**) BHJ active layer = 320 nm.

Figure 4 shows the calculated charge generation rate (in s^−1^∙cm^−3^) of SM BHJ OSCs obtained by the generalized transfer matrix method (GTMM). These calculated charge generation rate results provide a further explanation as to why a thicker junction of OSC has a higher *J*_sc_ value than a thinner junction of OSCs. From Figure 4a, the peak charge generation rate is located in the middle of the SM BHJ. In comparison with Figure 4b,c, the charge generation rate has the highest value in 100-nm-thick BHJ. However, under the thin junction circumstance, there is interference between the forward direction waves by absorption from the glass side and the backward direction waves by reflections from the electrodes in the photoactive layer. Therefore, the interference effect brings a low *J*_sc_ value in thin junction OSCs. By increasing the photoactive layer thickness containing BHJ, the OSC follows Beer-Lambert law. Therefore, the photo-generation rate is exponentially decreased by increasing the photoactive layer thickness.

**Figure 5:**
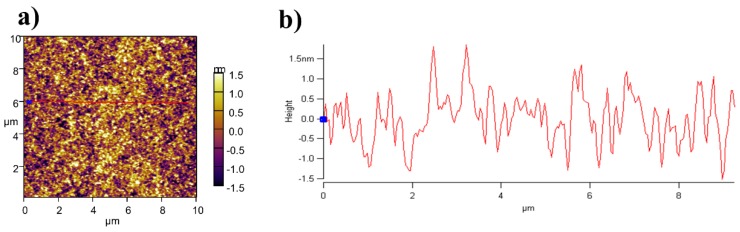
Measured atomic force microscopy (AFM) image of BHJ with a thickness of 320 nm: (**a**) top view of AFM; (**b**) measured cross-section of BHJ.

An AFM two-dimensional (2D) topography image of the *p*-DTS(FBTTh_2_)_2_:PC_70_BM BHJ film (thickness = 320 nm) is shown in Figure 5a. As reported, conjugated small molecules tend to aggregate strongly and form a crystalline structure [1,2,3,4,8,20]. Therefore, we assumed that the smaller domains in the device allow for a higher donor-acceptor interface area [1,2,3,4,8]. Finally, it has more efficient generations of charge carriers. From the literature, it has been suggested that below 15 nm, morphology control of the high efficiency of the small molecular solar cell is guaranteed [1,2,3,4,8]. In Figure 5b, it confirms that avoiding solvent treatment is effectively working in the control of the BHJ domains within 3 nm. Therefore, this would be strong and robust evidence of the morphology control of BHJ films without DIO solvent treatment.

## 4. Conclusions

In conclusion, we studied the high performance of small-molecule organic solar cells without additive solvent treatment on the control of the photoactive layer in nano-morphology. Significantly, we have obtained small-molecule organic solar cells with a comparable PCE of 7.22% by eliminating the DIO additional solvent treatment. Also, GTMM analysis was accomplished to confirm the effect of the thickness variations of SM BHJ OSCs. Lastly, AFM measurement also confirms that smaller domains have achieved efficient charge generation. These results provide significant progress in showing that solution-processed small-molecule organic solar cells without solvent treatment can be an alternative method to having a high-PCE device with polymeric and/or small-molecule counterparts.

## Figures and Tables

**Table 1:** A summary of SM BHJ OSC performances with changes of BHJ thickness. (PCE: Power conversion efficiency.)

BHJ Thickness	*V*_oc_ (V)	*J*_sc_ (mA/cm^2^)	Fill Factor	PCE (%)
100 nm	0.78	9.2	0.39	2.78
200 nm	0.80	11.1	0.41	3.64
320 nm	0.74	17.8	0.52	6.83
